# Immunological mechanisms and prevention strategies for febrile seizures in children

**DOI:** 10.3389/fimmu.2026.1833132

**Published:** 2026-07-10

**Authors:** Binbin Chen, Enfu Tao

**Affiliations:** 1 Department of Pediatrics, Wenling Maternal and Child Health Care Hospital, Wenling, Zhejiang, China; 2 Department of Neonatology and Neonatal Intensive Care Unit, Wenling Maternal and Child Health Care Hospital, Wenling, Zhejiang, China

**Keywords:** benzodiazepines, caregiver education, febrile seizures, HMGB1, neuroinflammation, NLRP3, prevention, risk stratification

## Abstract

Febrile seizures (FS) affect 2–5% of children globally, causing significant caregiver anxiety and healthcare utilization. Emerging evidence implicates neuroinflammation and T-cell-mediated immunity in FS pathogenesis, suggesting potential targets for future investigation. This Review synthesizes current evidence on FS prevention, emphasizing a paradigm shift from universal pharmacological approaches toward risk-stratified, personalized strategies. The COVID-19 pandemic provided unique insights: non-pharmaceutical interventions reduced FS incidence by 54–70%, while the Omicron variant emerged as a novel trigger associated with complex FS features. Prevention is conceptualized within a three-level framework: primary prevention targets all children through vaccination (MMR, PCV13, COVID-19 vaccines) and infection control; secondary prevention focuses on high-risk children with prior FS, where risk stratification integrates clinical predictors (complex features, young age, low fever), biomarkers (hyponatremia, zinc/vitamin D deficiency, inflammatory indices), and pathogen-specific risks (influenza A, Omicron); tertiary prevention addresses complications and epileptogenesis in children with complex FS or genetic predisposition (SCN1A, PCDH19). Key immunological mechanisms include HMGB1-NLRP3 inflammasome activation, TRPV1-mediated Th17 differentiation, and IL-1β/IL-10 dysregulation. Antipyretics do not prevent FS recurrence during distant febrile episodes, while intermittent benzodiazepines (diazepam, intranasal midazolam) effectively reduce early recurrence in high-risk children (NNT = 6.8), albeit with adverse effects in up to 36%. Emerging frontiers include novel therapeutic targets (HMGB1 inhibitors, TRP channel modulators, TSP-1 pathway inhibitors) and non-pharmacological innovations (wearable sensors, chronotherapy). Crucially, caregiver education underpins all prevention levels, addressing high rates of parental anxiety (58.2%). This integrated framework guides clinical practice toward more individualized, risk-based management.

## 1 Introduction

Febrile seizures (FS) represent the most prevalent convulsive disorder encountered in early childhood, constituting a significant source of anxiety for caregivers and a common reason for pediatric emergency consultations worldwide (1). The clinical spectrum of FS is defined by convulsive events occurring in association with a febrile illness, in the absence of an intracranial infection or other definable acute neurological cause, typically affecting children between 6 months and 5 years of age. The global prevalence of this condition is estimated to affect 2% to 5% of children, underscoring its substantial public health burden (2).

The peak incidence consistently occurs in the second year of life, with a notable male predominance observed in several cohorts (3, 4). While the majority of FS are self-limiting and carry an excellent prognosis, their occurrence imposes a considerable psychological toll on families and consumes significant healthcare resources. Understanding the etiology of the precipitating fever is central to contextualizing FS risk. Upper respiratory tract infections are the most frequently identified trigger, accounting for approximately half of all cases (3). Specific viral pathogens, however, carry differential risks. For instance, influenza virus infection is strongly associated with FS, with one Japanese database study reporting FS as a complication in 34.4% of hospitalized influenza cases in children under five, a rate dramatically higher than the 1.5% observed in hospitalized respiratory syncytial virus (RSV) cases (5). Conversely, human herpesvirus 6B (HHV-6B) has been specifically linked to complex febrile seizures (CFS), maintaining a stable disease burden even during periods when overall febrile illness rates declined (6).

The landscape of FS epidemiology has been dynamically reshaped by the COVID-19 pandemic, offering unique insights into prevention concepts. Stringent non-pharmaceutical interventions (NPIs) led to a marked reduction in common pediatric infections, which was paralleled by a significant decrease in FS incidence and seizure-related emergency department visits, particularly in the high-risk 0–6 years age group (4, 7). This observed correlation strongly suggests the utility of broad infection control measures as a population-level preventative strategy. However, the emergence of the SARS-CoV-2 virus itself introduced a new pathogenic trigger. Notably, FS were almost exclusively associated with the Omicron variant, which has been linked to a higher proportion of complex FS features compared to non-COVID-19 FS (8, 9).

Beyond infectious triggers, emerging evidence has implicated neuroinflammation and immune dysregulation as key contributors to FS pathogenesis (10, 11). Children with FS exhibit elevated levels of pro-inflammatory cytokines, activation of the NLRP3 inflammasome, and dysregulated T-cell responses (12, 13). Notably, the high-mobility group box 1 (HMGB1)-NLRP3 inflammasome pathway, febrile temperature-driven TRPV1-mediated Th17 differentiation, and altered IL-1β/IL-10 balance have been identified as potential molecular mechanisms linking fever to seizure susceptibility (10, 12, 13). These immunological insights not only deepen our understanding of FS pathophysiology but also open new avenues for mechanism-based prevention strategies.

Recurrence is a core concern in FS management, with reported rates varying across populations (1, 14). Factors influencing recurrence include a younger age at first seizure, a family history of FS, and the occurrence of complex features. Notably, developmental delays appear more common in children experiencing recurrent FS, hinting at potential underlying susceptibilities that warrant further investigation (1). The immediate burden of an FS episode, coupled with the anxiety surrounding potential recurrence and the rare but serious risk of progression to febrile status epilepticus, frames the imperative for effective prevention strategies (8, 15). Prevention in this context must be conceptualized broadly, encompassing primary prevention of the initial seizure, secondary prevention of recurrence, and tertiary prevention of complications and long-term sequelae. This conceptual framework extends beyond pharmacological interventions to include caregiver education, judicious use of antipyretics, targeted prophylaxis in high-risk individuals, and public health measures such as vaccination and infection control.

This review aims to synthesize current evidence and emerging perspectives on the prevention of FS in children. It will navigate the foundational clinical management guidelines, critically appraise pharmacological strategies including antipyretics and rescue therapies, and explore the critical role of risk stratification in personalizing care. By integrating insights from recent epidemiological shifts, including those observed during the COVID-19 pandemic, this review seeks to provide a comprehensive overview of the multi-faceted approach required to mitigate the burden of FS, ultimately aiming to guide clinical practice and inform future research directions towards optimized, evidence-based preventative paradigms.

## 2 Search strategy and selection criteria

In this narrative review, we conducted a comprehensive search of PubMed, Web of Science, Embase, and the Cochrane Library for articles published between January 2020 and March 2026. The search strategy employed combinations of the following keywords: “febrile seizures,” “febrile convulsions,” “pediatric,” “children,” “prevention,” “prophylaxis,” “immunology,” “neuroinflammation,” “risk factors,” “biomarkers,” “vaccination,” “antipyretics,” “benzodiazepines,” “caregiver education,” and “long-term outcomes.” We prioritized the inclusion of systematic reviews, meta-analyses, randomized controlled trials, and large prospective cohort studies to underpin clinical recommendations. Preclinical studies, exploratory reports, and research involving animal models were incorporated only when they offered essential mechanistic insights into pathogenesis or identified emerging therapeutic targets not accessible through clinical studies alone. Additionally, the reference lists of selected articles and pertinent clinical practice guidelines were manually reviewed to identify further relevant references. Only articles published in English were considered for inclusion. In order to convey the level of certainty regarding evidence across various outcome domains, we employed the principles of the Grading of Recommendations Assessment, Development, and Evaluation (GRADE) framework as a conceptual framework to organize our discussion on the strength of evidence, rather than conducting a formal grading assessment (16).

## 3 Foundations of clinical management: guidelines, definitions, and acute care pathways

A coherent and evidence-based clinical management framework is paramount for the effective and safe handling of FS, a condition that, while generally benign, frequently triggers emergency consultations and parental distress (17). The foundation of this management rests on precise definitions, adherence to established guidelines, and the implementation of standardized acute care pathways aimed at accurate diagnosis, appropriate intervention, and the avoidance of unnecessary investigations. The definition of a FS is universally acknowledged as a seizure occurring in association with a fever (temperature ≥38 °C or 100.4°F) in a child aged between 6 months and 5 years, without evidence of an intracranial infection or a defined cause, and in the absence of a history of prior afebrile seizures (18, 19). This core definition immediately distinguishes FS from acute symptomatic seizures due to meningitis or encephalitis and from epilepsy. A critical subsequent distinction is between simple febrile seizures (SFS) and CFS. SFS are generalized, last less than 15 minutes, and do not recur within 24 hours or within the same febrile illness. In contrast, CFS are defined by features such as a focal onset or presentation, duration exceeding 15 minutes, or multiple occurrences within 24 hours (20). This classification is not merely academic; it directly informs diagnostic urgency, management intensity, and prognostic considerations (17, 20).

Despite the availability of numerous national and international guidelines published over the past three decades, a systematic review reveals both significant convergence and notable variations in their recommendations (21). Commonalities include the general discouragement of routine neuroimaging and lumbar puncture (LP) for children with SFS who have returned to their neurological baseline, and the strong recommendation against long-term antiepileptic drug prophylaxis for SFS due to their benign natural history and the potential side effects of medications (17, 21). However, disparities exist, particularly regarding criteria for hospital admission, the specific indications for performing an LP, and the management of CFS (21). For instance, admission criteria often include young age (e.g., <18 months) and complex features, but the exact thresholds can vary (21). These guideline differences can translate into heterogeneous clinical practices. A nationwide survey in Greece highlighted that a substantial proportion of pediatricians admitted to employing non-evidence-based practices, such as suggesting aggressive fever management at low temperatures or systematic referral to a specialist after any FS episode, even while acknowledging these practices’ lack of efficacy (22). This gap between guideline recommendations and real-world application underscores the need for continuous education and the implementation of standardized clinical pathways (18, 22).

The acute care pathway for a child presenting with a FS begins with immediate stabilization, focusing on airway, breathing, and circulation, followed by a rapid but thorough clinical assessment. The primary goal is to identify the cause of the fever and, crucially, to exclude serious underlying conditions like bacterial meningitis or intracranial pathology. For the child with a typical SFS who is alert and back to baseline after the post-ictal period, extensive diagnostic testing is not warranted. Multiple studies and guidelines affirm that routine laboratory tests, neuroimaging, and LP have a very low yield in this population (18, 20, 23). A large US study of children’s hospitals demonstrated a significant decline in the utilization of LPs, head CT scans, complete blood counts, and hospital admissions for SFS over a 15-year period, with no concomitant increase in missed cases of bacterial meningitis, strongly supporting a conservative approach (23). This trend towards less invasive management is a cornerstone of modern FS care, aiming to reduce healthcare costs, avoid unnecessary patient discomfort, and minimize parental anxiety (21, 23).

Nevertheless, clinical judgment remains essential. The decision to perform an LP must be individualized, considering factors such as the child’s age, immunization status, clinical signs of meningitis, and the presence of complex features (19, 24). While the prevalence of bacterial meningitis in children with FS is low, evaluation for possible meningitis remains a necessary aspect of management, particularly in regions with different epidemiological patterns or in the context of specific vaccinations (24). For example, aseptic meningitis following measles, mumps, and rubella (MMR) vaccination should be considered in the differential diagnosis (24). The emergence of new pathogens also influences this assessment; during the SARS-CoV-2 Omicron wave, FS were a common neurological manifestation in hospitalized children, though typically mild and self-limiting, necessitating awareness but not necessarily altering the fundamental diagnostic approach for the seizure itself (25).

The management of CFS and febrile status epilepticus (FSE) constitutes a more urgent and resource-intensive branch of the acute care pathway. FSE, defined as a FS lasting 30 minutes or more, is the most common form of status epilepticus in young children (26, 27). While the prognosis for cognitive outcomes in FSE is generally favorable, appropriate and timely termination of the seizure is critical to prevent morbidity. Treatment protocols have evolved to improve outcomes. A historical cohort study showed that the implementation of a structured treatment protocol for FSE, compared to discretionary management, was associated with a lower rate of poor neurological outcomes (26). First-line treatment universally involves benzodiazepines. For seizures persisting beyond this, second-line agents like fosphenytoin or phenobarbital are used, with progression to anesthetic agents (barbiturate coma therapy) for refractory cases (26). Recent data-driven analyses have helped delineate clinical subgroups within pediatric status epilepticus. One distinct cluster comprises younger patients with acute status epilepticus associated with infection, including febrile status epilepticus, characterized generally by a good response to treatment and favorable outcomes (27). This clustering can aid in prognostication and resource allocation. Risk factors for progression to or recurrence of status epilepticus after a first seizure include young age (under 1 year), pre-existing neurological abnormalities, and discontinuation of antiepileptic treatment, whereas a SFS is identified as a protective factor (28).

A key decision point in the emergency department (ED) is determining the appropriate observation period prior to discharge. The rationale for observation is to monitor for seizure recurrence, which could indicate a more serious condition or require further intervention. However, evidence suggests that the risk of short-term recurrence after a SFS is low. One study found that recurrence occurred in only 1.9% of children, and all recurrences happened within the first 3 hours of presentation to the ED (29). Similarly, a study on afebrile seizures, which provides a relevant context, found that 86% of recurrences happened within the first 6 hours (30). These findings challenge the necessity of prolonged ED observation for children with SFS who have returned to their neurological baseline. Consequently, a growing consensus supports that stable children may be considered for earlier discharge after appropriate caregiver education is provided, thereby reducing ED crowding and healthcare costs without compromising safety (29).

The disposition of the child—whether discharged home, observed, or admitted—integrates all preceding assessments. Hospital admission is not routinely required for SFS. Indications for admission may include the need for further evaluation of the febrile illness, concerns about the family’s ability to manage or return for care, very young age, or features of a CFS, though the systematic admission of CFS patients is also being questioned as often unnecessary (20). The role of urgent electroencephalogram (EEG) in the ED is limited. An EEG is typically not indicated after a SFS. Its utility is higher in cases of atypical or complex presentations, focal deficits, or prolonged alteration of consciousness. An observational study found that nearly a quarter of patients with atypical FS showed EEG abnormalities, whereas EEG was uniformly normal in cases of suspected syncope, guiding differential diagnosis (31). We acknowledge that the diagnostic and management recommendations in this section are largely based on evidence from high-resource settings, as reflected in the guidelines cited above. In resource-limited environments where diagnostic testing may be constrained, clinical judgment should guide management adapted to local resources. A detailed comparison of key recommendations from major guidelines is summarized in **Table 1**.

**Table 1:** Comparison of key recommendations from major febrile seizure guidelines.

Clinical question/intervention	AAP (USA)	NICE (UK)	Italian guideline (LICE)	Consensus areas	Discrepancies/key variations	Supporting references
Routine Lab Tests for Simple FS	Not recommended.	Not recommended.	Not recommended.	Strong consensus against. No value in otherwise well child.	—	(18, 21, 23)
Routine Neuroimaging (CT/MRI) for Simple FS	Not recommended.	Not recommended.	Not recommended.	Strong consensus against.	—	(18, 21, 23)
Routine Lumbar Puncture for Simple FS	Not recommended if child returns to baseline and has no meningeal signs. Key exceptions: Unimmunized/under immunized, pretreated with antibiotics, immunocompromised.	Not recommended in a well-appearing child without meningeal signs.	Not recommended in a well-appearing child without meningeal signs.	Consensus against routine use.	AAP provides specific age-based guidance (<12, 12-18, >18 months) and lists explicit exceptions. NICE & LICE rely solely on clinical signs.	(19, 21, 24)
Routine EEG for Simple FS	Not recommended.	Not recommended.	Not recommended.	Strong consensus against.	All agree it may be considered in complex FS or children with developmental abnormalities.	(16, 31)
Routine Hospital Admission for Simple FS	Not recommended.	Not recommended.	Not recommended.	Strong consensus against.	Social factors (parental anxiety, distance from care), prolonged post-ictal state may justify individual admission.	(20, 21)
Long-term Antiepileptic Drug Prophylaxis for Simple FS	Strongly recommended AGAINST.	Strongly recommended AGAINST.	Strongly recommended AGAINST.	Strong consensus against. Risks (side effects) far outweigh any potential benefit.	—	(17, 21, 32)
Management of Complex FS	Individualized. Consider observation/admission based on clinical assessment.	Individualized.	Individualized.	Consensus on individualized approach. No routine protocol.	Historical guidelines recommended routine admission. Modern practice is to assess risk of CNS infection/seizure recurrence to guide need for admission/workup.	(20, 21)
Lumbar Puncture Indications (Detailed)	<12 months: Consider routinely. 12–18 months: Individualize. >18 months: Only if meningeal signs or other red flags.	Perform if meningeal signs or impaired consciousness are present.	Perform if child appears toxic or has meningeal signs.	Partial consensus: All agree on LP for clear meningeal signs.	Major variation in age-based vs. purely symptom-based thresholds. AAP’s age-cutoff approach is unique.	(19, 21, 24)
Intermittent Benzodiazepine Prophylaxis (e.g., Diazepam)	Not recommended for routine use. May be considered for a select few children at very high risk (e.g., prolonged prior FS) after thorough discussion of limited benefits vs. frequent side effects.	Not routinely recommended.	Not recommended for routine use. May be considered in selected high-risk cases.	Consensus against routine use.	NICE is most conservative (not routinely recommended). AAP and LICE leave a narrow door open for extreme individual cases but emphasize it is NOT standard care.	(21, 32)
Post-Discharge Follow-up & Parental Education	Recommended. Provide information on recurrence, first-aid, and reassurance.	Recommended.	Recommended.	Strong consensus FOR. Cornerstone of management to reduce anxiety and unnecessary care.	—	(17, 33)

AAP, American Academy of Pediatrics; NICE, National Institute for Health and Care Excellence (UK); LICE, Italian League Against Epilepsy; FS, febrile seizure; CT, computed tomography; MRI, magnetic resonance imaging; EEG, electroencephalogram; LP, lumbar puncture; CNS, central nervous system. Core Message: Guidelines show remarkable consistency in de-escalating care for simple FS (no tests, no admission, no drugs). The main variations lie in the interpretation of risk for LP and the extremely narrow role (if any) for drug prophylaxis. Educating and reassuring caregivers is universally endorsed as the most critical intervention.

## 4 Pharmacological strategies for seizure recurrence prevention: antipyretics, rescue, and prophylactic benzodiazepines

Building on the foundation of acute management and caregiver education, the consideration of pharmacological agents to prevent FS recurrence represents a nuanced clinical decision. The choice to intervene pharmacologically must be carefully weighed against the generally benign prognosis of most FS, the potential for adverse drug effects, and the overarching goal of minimizing disruption to the child’s and family’s quality of life. The primary pharmacological strategies can be categorized into three broad approaches: the use of antipyretics to modify the febrile stimulus, the intermittent or “rescue” use of benzodiazepines at the onset of subsequent febrile illnesses, and the continuous administration of prophylactic antiepileptic drugs. Crucially, current evidence and clinical practice guidelines strongly recommend against routine pharmacological prophylaxis for the vast majority of children with FS. The role of any agent is strictly limited to a very select, high-risk minority after a thorough discussion of risks and benefits. A comprehensive overview of these strategies is provided in **Table 2**.

**Table 2:** Pharmacological and non-pharmacological interventions for febrile seizure prevention: efficacy, safety, and clinical recommendations.

Intervention	Regimen	Efficacy (recurrence risk)	NNT/NNH	Major adverse effects	Adverse effect rate	Recommendation level & rationale	Target Population	References
Caregiver Empowerment and Education	Structured discharge education + psychological support + provision of emergency contact.	No direct effect on recurrence rate. Indirect benefit: Reduces parental anxiety, improves correct acute management.	N/A	No direct effect on recurrence rate. Indirect benefit: Reduces parental anxiety, improves correct acute management.	0%	Strong recommendation FOR all children with FS. Cornerstone of management.	All FS children and their caregivers.	(33, 35–38)
Antipyretics (acetaminophen/ibuprofen)	As needed during fever for comfort	Distant fever episodes: No benefit (OR 0.92, 95% CI 0.57-1.48). Same febrile illness: Possible benefit (OR 0.33, 95% CI 0.19-0.57; Low-certainty evidence).	N/A	Minimal at standard doses.	<5%	Strong recommendation AGAINST use for prophylaxis of future FS. May be considered during the same illness based on weak evidence, primarily for fever comfort.	All febrile children (for symptom relief, not seizure prevention).	(32, 39)
Intermittent Diazepam	Rectal or oral at fever onset (e.g., every 8h).	Effective: At 6 months: RR 0.64 (95% CI 0.48-0.85) At 24 months: RR 0.73 (95% CI 0.56-0.95)	NNTB~5-14 (for long-term recurrence)	Sedation, ataxia, agitation, paradoxical hyperactivity.	Up to ~30%	Not recommended for routine prophylaxis. May be considered on an individual basis for a select few children at very high risk (e.g., history of prolonged FS, high parental anxiety) after thorough discussion of limited benefits vs. frequent side effects.	Very select high-risk children (not for most children with FS). Decision requires shared decision-making.	(32, 40, 41)
Intermittent Midazolam	Intranasal/buccal at fever onset.	Evidence for prophylaxis is lacking. Shown to be effective for acute seizure termination (comparable to IV diazepam).	N/A (for prophylaxis)	Sedation, local irritation.	~30% (from acute treatment studies)	Evidence insufficient to recommend for FS prophylaxis. Recommended as a non-IV rescue medication for acute seizure clusters (not for prevention).	Children requiring rescue therapy for acute repetitive seizures.	(42, 43)
Intermittent Clobazam	Oral at fever onset.	Significant reduction at 6 months in one trial, but control group recurrence was 83.3%. Needs replication.	Not calculated	Sedation.	~30%	Weak/Insufficient evidence. Not recommended for routine use. More research needed.	Potential alternative in research settings if intolerant to diazepam.	(32)
Intermittent Levetiracetam	Oral at fever onset.	Significant reduction at 12 months in a single study.	Not calculated	Sedation, irritability.	~20%	Exploratory (Very low certainty). Not recommended for clinical use outside of research.	Research setting only.	(32)
Continuous Phenobarbital	Daily oral.	Effective: RR ~0.5-0.7.	NNTB~8-10	Cognitive impairment, behavioral disturbances (hyperactivity, irritability), sleep disorders.	30-40%	Strong recommendation AGAINST use for FS prophylaxis. Adverse effects are common and significant, outweighing the benefit for a benign condition.	Should not be used for FS. Reserved for children with comorbid epilepsy.	(32)
Continuous Valproate	Daily oral.	No significant benefit versus placebo/no treatment.	N/A	Hepatotoxicity, thrombocytopenia, weight gain.	10-20%	Strong recommendation AGAINST use. No proven efficacy for FS prophylaxis.	Not applicable for FS prophylaxis.	(32)
Caregiver Empowerment & Education	Structured discharge education + psychological support + provision of emergency contact.	No direct effect on recurrence rate. Indirect benefit: Reduces parental anxiety, improves correct acute management, decreases unnecessary healthcare utilization.	N/A	None.	0%	Strong recommendation FOR all children with FS and their families. Cornerstone of management. Provide information on FS nature, first-aid, reassurance of benign prognosis, and when to seek medical help.	All FS children and their caregivers.	(33, 34, 36–38)

NNT, number needed to treat; NNTB, number needed to treat for an additional beneficial outcome; NNH, number needed to treat for an additional harmful outcome; RR, risk ratio; CI, confidence interval; FS, febrile seizure; N/A, not applicable. Key Takeaway: The evidence supports against the routine use of any drug for FS prophylaxis due to frequent side effects and the condition’s benign outcome. Educating and reassuring caregivers is the most important and universally recommended intervention.

The intuitive use of antipyretics to prevent FS by reducing the core febrile trigger has been a mainstay of parental practice, yet robust clinical evidence does not support its efficacy for preventing *distant* FS episodes. A comprehensive systematic review and meta-analysis by Hashimoto et al. concluded that there is clearly no role for antipyretic prophylaxis in preventing FS during distant fever episodes, with an odds ratio of 0.92 (95% CI, 0.57–1.48) from randomized controlled trials (39). This finding is corroborated by the updated Cochrane Review, which found no significant benefit for ibuprofen or diclofenac versus placebo or no treatment in preventing recurrence (32). The biological rationale that fever lowering per se prevents seizures appears insufficient, as the seizure threshold in susceptible children may be crossed even with modest temperature elevations. However, a more nuanced question pertains to the prevention of multiple seizures within the same febrile illness, a scenario with a baseline risk estimated at 14-20% (44). The same systematic review noted weak evidence suggesting a possible role for antipyretics in this specific context, citing one study where acetaminophen reduced recurrence within the same episode (9.1% vs. 23.5% in controls) (39). This potential window of effect underscores the importance of intervention timing, but the overall evidence remains very limited and does not justify the routine prophylactic use of antipyretics for FS prevention. Their primary role remains improving child comfort during febrile illnesses.

Intermittent diazepam is the most extensively studied agent for FS prophylaxis. The Cochrane Review provides moderate- to high-certainty evidence that intermittent diazepam, compared to placebo or no treatment, significantly reduces recurrent FS at multiple time points, with risk ratios ranging from 0.64 at six months to 0.73 at 24 months (32). This preventive effect is most salient for reducing recurrences within the same febrile illness or during subsequent illnesses in the near term. A prospective observational study by Tanaka et al. directly compared diazepam suppositories combined with as-needed acetaminophen versus acetaminophen alone for preventing recurrence during the same fever episode in children with suspected simple FS (40). The recurrence rate was significantly lower in the diazepam group (3.5% vs. 12.2%), yielding an adjusted odds ratio of 0.23, without an increase in severe adverse events or medical costs (40). This real-world data supports the efficacy of rescue benzodiazepines in a targeted setting. The clinical impact of withholding this intervention was starkly illustrated in a retrospective study following a revision to Japanese FS guidelines that reduced prophylactic diazepam use (45). After the guideline change, rectal diazepam administration decreased from 53% to 17%, which was associated with a significant increase in seizure recurrence within 24 hours from 12% to 20%. Multivariable analysis identified prophylactic diazepam as the sole factor related to preventing early recurrence, with a relative risk reduction of 70% and a number needed to treat of 6.8 (45). However, given that adverse effects occur in up to 30% of children receiving diazepam, routine prophylaxis is not recommended. Diazepam may be considered only in a very select population of high-risk children (e.g., those with a history of prolonged FS or febrile status epilepticus) following a thorough discussion of the limited benefits versus frequent risks.

The practical application of intermittent benzodiazepine therapy has evolved significantly with the introduction of non-rectal formulations, primarily intranasal products, which enhance social acceptability and ease of administration. Historically, rectal diazepam gel was the primary rescue medication for out-of-hospital use. However, limitations related to administration route have driven a rapid transition. A large database study documented a shift from rectal diazepam to intranasal midazolam or intranasal diazepam (42). This transition was associated with a significant increase in both the average wholesale price and the out-of-pocket cost to patients (42). Despite the higher cost, the improved convenience and likely faster administration in a stressful situation are considered major advantages. Prescription patterns for home rescue benzodiazepines are not uniform. In a cohort of over 80,000 children with FS, only about 12% filled a prescription for a non-intravenous rescue benzodiazepine (46). The factors most strongly associated with receiving a prescription were CFS (OR: 3.51), initial inpatient hospitalization for the FS (OR: 3.53), recurrent FS, and an eventual diagnosis of epilepsy (46). This indicates that clinicians are generally reserving rescue therapy for a minority of children perceived to be at higher risk or with more severe presentations. Midazolam occupies a distinct role from diazepam, functioning primarily as a first-line agent for the acute termination of FS rather than for long-term prophylaxis (47, 48). A prospective randomized controlled trial demonstrated that midazolam is as safe and effective as diazepam for managing FS, while offering a key practical advantage: the mean time from hospital arrival to treatment initiation was significantly shorter in the midazolam group (47). Another comparative study found intranasal midazolam to be as safe and effective as intravenous (IV) diazepam, though seizure control was achieved more quickly with the IV route (47). Its favorable pharmacokinetic profile—including rapid absorption and a short half-life—supports its use via multiple, socially acceptable routes (intranasal, buccal, intramuscular), making it a preferred option when IV access is not readily available. This versatility is reflected in clinical protocols and real-world practice. A review of U.S. state-level protocols found that 44.1% of states specifically recommended midazolam preferentially over other benzodiazepines, and the NASEMSO guidelines recommend intranasal or intramuscular midazolam unless vascular access is already established (48). A Belgian guideline similarly recommends buccal midazolam as the first step when IV access is unavailable, with weight-based dosing (49). However, while midazolam is unequivocally effective for acute seizure termination, there is insufficient evidence to support its use for the prevention of future FS episodes. Its role is therefore strictly limited to acute rescue therapy in the context of FS, rather than long-term prevention. A comprehensive overview of these roles is provided in **Table 2**.

Levetiracetam has emerged as an important agent, serving both as a second-line acute treatment and as a potential prophylactic option (32, 47). This positions it as a key complement to benzodiazepines in the FS management spectrum. For acute seizure management, levetiracetam (60 mg/kg IV) is increasingly recommended as a second-line anti-seizure medication when first-line benzodiazepines fail, as outlined in clinical protocols (30, 49). Its use is driven by a favorable safety profile and the convenience of administration without the need for therapeutic drug monitoring. For prophylactic management, a randomized controlled trial in China demonstrated that intermittent oral levetiracetam significantly reduced FS recurrence compared to a control group over 48 weeks (RR 0.27, 95% CI 0.15 to 0.52), with a number needed to treat of 3 (50). The study reported a good safety profile, with only one patient in the levetiracetam group exhibiting severe drowsiness. The Cochrane Review highlights this single trial, noting that while levetiracetam shows benefit, the certainty of evidence is very low due to small sample size and risk of bias, and further research is required (32). Therefore, levetiracetam should be considered an exploratory or research-stage option and is not recommended for routine clinical practice at this time. Its primary role in FS management is as a second-line acute treatment, rather than as a first-line prophylactic agent.

The safety and tolerability profile of benzodiazepines is a critical consideration in their prophylactic use. Adverse effects, notably sedation, ataxia, and paradoxical agitation, are common but typically mild and transient. The Cochrane Review notes that adverse effects were recorded in up to 36% of children in benzodiazepine-treated groups (32). The study by Tanaka et al. observed mild ataxia significantly more often in the diazepam group (29.4% vs. 18.7%) but no severe adverse events (40). These effects, while often mild, must be weighed against the significant clinical impact in daily practice: a substantial proportion of children experience these side effects, which can lead to parental anxiety, medication non-adherence, and reduced quality of life for the child, particularly during febrile illnesses when medication is given. This burden is a key reason why prophylaxis is reserved for a carefully selected high-risk minority. Therefore, the primary management strategy for the vast majority of children shifts decisively away from universal medication toward structured caregiver education as the cornerstone of care. The role of intermittent benzodiazepines is refined to a targeted, rescue intervention for children at highest risk of prolonged or clustered seizures (e.g., those with a history of CFS, young age, or intractable parental anxiety after thorough education). The choice of agent involves practicalities; while diazepam has a long history of use and evidence, other options exist. Midazolam, frequently used intranasally, is favored in many settings for its rapid absorption and shorter half-life. One trial included in the Cochrane Review found a significant benefit for intermittent clobazam compared to placebo at six months, though this effect was observed against an extremely high (83.3%) recurrence rate in the control group, necessitating replication (32). The decision to employ these agents ultimately hinges not just on clinical factors, but also on local healthcare infrastructure, medication accessibility, cost, and—fundamentally—the level of parental anxiety after thorough education and reassurance (44).

Emerging evidence points to other agents that may hold promise. The Cochrane Review highlighted one trial where intermittent oral levetiracetam significantly reduced recurrent seizures at 12 months compared to placebo, with a good safety profile, though the certainty of evidence was very low and further study is required (32). This aligns with a broader understanding of FS pathogenesis involving neuroinflammation and neuronal hyperexcitability, suggesting potential future targets beyond traditional GABAergic modulation (51). As research progresses on the interplay of fever, genetic susceptibility, and neuroinflammation, novel therapeutic targets may emerge (51). However, at present, intermittent benzodiazepines remain the pharmacological cornerstone for recurrence prevention in high-risk scenarios. The decision to employ them hinges not just on clinical factors like seizure complexity and recurrence history, but also on local healthcare infrastructure, medication accessibility, cost, and, fundamentally, the level of parental anxiety after thorough education and reassurance. This sets the stage for a more detailed exploration of how to identify which children are most likely to benefit from such targeted pharmacological strategies.

## 5 Risk stratification and prediction: identifying candidates for targeted prevention

The decision to employ pharmacological strategies for FS prevention, as discussed previously, hinges critically on the ability to identify children at the highest risk for recurrence. A blanket preventive approach is neither practical nor advisable, making accurate risk stratification the cornerstone of personalized management. This involves a multifaceted assessment that integrates well-established clinical predictors, emerging biomarker data, and an understanding of specific triggering pathogens to refine prognostic models and guide targeted interventions. These risk factors are systematically synthesized in **Table 3**, which provides a structured overview stratified by evidence level and clinical utility, serving as a practical reference for clinicians.

**Table 3:** Risk stratification for febrile seizure recurrence and adverse outcomes.

Risk category	Specific factor	Same-illness recurrence	Long-term recurrence	Epilepsy risk	Clinical application	References
Demographics	Age <12 months	✓	✓✓	✓	Assess long-term risk after first FS	(52, 53)
	Age 12–18 months	—	✓	—	Lower risk than <12 months	(52, 53)
	Male sex	✓	—	—	Predicts early recurrence	(3, 14, 54)
	Developmental delay/neurological abnormality	—	✓	✓✓	Requires neurology follow-up	(1, 51)
Clinical Features	First seizure duration >15 minutes	—	✓	✓✓	Core complex FS criterion	(51, 53)
	Focal onset	—	✓	✓✓	Neuroimaging indicated	(20, 51)
	Multiple seizures within 24 hours	✓✓	✓	✓	Strongest predictor of early recurrence	(20, 41, 44)
	First seizure temperature <38.5 °C	✓✓	✓✓	—	Key predictor of same-illness recurrence	(41, 53, 54)
	First seizure temperature 38.5-39.8 °C	✓	✓	—	Intermediate risk	(41, 54)
Family History	First-degree relative with FS	✓	✓✓	—	Assess in all FS children	(1, 52, 55)
	First-degree relative with epilepsy	—	—	✓✓	Core epilepsy risk assessment	(51)
Laboratory Biomarkers	Hyponatremia (<135 mmol/L)	✓	✓	✓	Recommended in complex FS	(56, 57, 59, 61, 71)
	Hypozincemia	✓	✓✓	—	Consider supplementation, moderate evidence	(56, 58, 71)
	Vitamin D deficiency (<20 ng/mL)	—	✓✓	—	Recurrence risk assessment	(56, 59)
	Anemia/iron deficiency	✓	—	—	Modifiable risk factor	(60)
	NLR >2.5	✓	—	—	Research stage, clinical reference	(61, 62)
	Elevated SII	✓	—	—	Research stage	(61)
Pathogen-Specific	Influenza A	✓✓	—	—	High risk for same-illness recurrence	(63, 64)
	Influenza B	✓	—	—	Lower risk than influenza A	(63, 64)
	HHV-6B	—	—	✓ (complex FS)	Associated with complex FS	(7)
	Omicron variant	✓	—	—	Higher proportion of complex FS	(9, 65)
	Adenovirus	✓	—	—	General risk	(66)
Environmental/Behavioral	Non-breastfeeding	—	✓	—	Protective: breastfeeding OR 0.65	(67, 68)
	Daycare attendance	✓	—	—	Increased infection exposure	(66)

FS, febrile seizure; NLR, neutrophil-to-lymphocyte ratio; SII, systemic immune-inflammation index; HHV-6B, human herpesvirus 6B; OR, odds ratio. Note: ✓ indicates association; ✓✓ indicates strong/predictive association; — indicates no consistent evidence or not applicable. Evidence grades based on study design and consistency across meta-analyses.

The risk of FS recurrence is stratified into two primary timelines: early recurrence within the same febrile illness (often within 24 hours) and long-term recurrence during subsequent febrile episodes. For early recurrence, several clinical factors have been consistently identified. A history of a previous early recurrent FS episode is a potent predictor, with one study reporting an odds ratio of 10.161 for recurrence within 24 hours (41). Notably, a lower body temperature at the time of the initial seizure is also significantly associated with early recurrence (41, 54). One analysis found that the combination of male sex and a body temperature ≤ 39.8 °C had a high sensitivity and negative predictive value for identifying children at risk for a recurrent seizure during the same illness (54). Furthermore, a prior history of FS in general and a family history of FS are independent risk factors for acute-phase recurrence (63). The causative pathogen also plays a role; influenza A infection has been specifically identified as a key independent risk factor for acute-phase recurrence (63). The timing of recurrence is also predictable, with the vast majority (82-95%) of early recurrent seizures occurring within 8 to 24 hours of the first episode (63, 69), informing the critical window for observation or prophylactic intervention.

For long-term recurrence over subsequent years, a different set of predictors emerges. Younger age at the first FS episode is consistently one of the most powerful risk factors (52, 53). A systematic review confirms that a positive family history of FS, particularly in first-degree relatives, significantly increases recurrence risk (55). Contrary to intuition, a lower peak body temperature during the febrile illness is associated with a higher recurrence risk (53). The duration of the first seizure is also an independent predictor, with longer initial episodes correlating with increased recurrence odds (53). Beyond these classic factors, other demographic and clinical features such as diurnal variation of the initial seizure and gender have been incorporated into predictive models (52). Novel multivariate analyses also suggest that factors like height percentile and hemoglobin concentration may be linked to FS recurrence, indicating a potential role for nutritional and growth parameters (70).

The search for objective, measurable biomarkers to augment clinical prediction is an active area of research. Serum electrolyte imbalances, particularly hyponatremia, have been repeatedly associated with FS occurrence, complexity, and recurrence (56). Trace element deficiencies are also strongly implicated. Meta-analytic evidence confirms that children with FS have significantly lower serum levels of zinc, selenium, and magnesium compared to febrile or healthy controls (58). Individual studies further detail these relationships: lower zinc levels are associated with both the occurrence of FS and an increased risk of complex seizures (57, 71), while low serum sodium and potassium are also risk factors for complex seizures (57). Vitamin D status is another area of focus, with deficiency and insufficiency being common in children with SFS and showing a significant negative correlation with the frequency of recurrent episodes (59). A comprehensive prospective study identified low sodium, low vitamin D, and low zinc as independent risk factors for FS, with low zinc specifically associated with seizure recurrence (56).

Inflammatory and hematological indices, being readily accessible, offer practical predictive value. Markers like the neutrophil-to-lymphocyte ratio (NLR), systemic immune-inflammation index (SII), and systemic inflammation response index (SIRI) are significantly elevated in children with FS compared to febrile and healthy controls, suggesting their potential utility as diagnostic aids (61). While these indices may not reliably distinguish between simple and complex FS types (62), they reflect the systemic inflammatory state associated with seizure genesis. Furthermore, iron deficiency and anemia have been meta-analytically associated with an increased susceptibility to FS, with poor iron indices (low mean corpuscular volume, serum iron, and ferritin) serving as incremental risk factors (60). However, most of these biomarker associations are observational, with modest effect sizes, a lack of standardized cut-offs, and limited external validation. Therefore, these markers remain investigational and are not ready for routine clinical implementation.

The nature of the underlying infection is a major determinant of FS risk and phenotype. Ecological and cohort studies robustly link specific viruses to increased FS incidence. Influenza virus, particularly influenza A, is a well-established high-risk pathogen, with infection leading to high fever that can trigger FS in susceptible children; it is associated with both a higher frequency of neurologic complications including FS and an increased risk of early recurrence (63, 64). The winter peak in FS incidence is statistically attributable to circulating viruses like influenza, RSV, and human metapneumovirus (66). The emergence of SARS-CoV-2, particularly the Omicron variant, introduced new characteristics. Children with COVID-19 presenting with FS are often older, exhibit longer seizure durations, and have a higher proportion of complex FS features, including cluster seizures and status epilepticus, compared to those with non-COVID-19 FS (8, 65). Specific inflammatory patterns, such as lymphopenia and elevated creatine kinase, are also noted in COVID-19-associated FS (65). Furthermore, in the context of COVID-19, risk factors for more severe neurological outcomes like acute encephalopathy include older age (over 3 years), cluster seizures, status epilepticus, and specific laboratory findings like hyperglycemia and metabolic acidosis (72, 73). A history of FS and decreased NK cell count have been identified as independent risk factors for convulsions in children with COVID-19 (74).

Integrating these diverse risk factors into clinically usable tools is the next step. Several studies have developed and validated prediction models. Nomograms incorporating predictors such as young age at first FS, family history of FS, diurnal variation, gender, and C-reactive protein level have demonstrated good performance for predicting long-term recurrence (52). However, this model was derived from a single-center cohort and lacked external validation in geographically diverse populations, limiting its generalizability. Another model focused on the transition from FS to epilepsy identified lower sodium, elevated red cell distribution width (RDW) and interleukin-6 (IL-6), and specific EEG findings (background slow rhythm and epileptiform discharges) as key predictive factors (75). While this nomogram showed favorable discrimination, its reliance on EEG findings—which are not routinely performed in all clinical settings—may restrict its practical applicability. For predicting the initial occurrence of FS in a febrile child, models based on complete blood count parameters have shown high predictive ability (AUC 0.858–0.884) (76). Despite strong internal validation, the specificity of this model (0.72–0.78) suggests a notable proportion of false positives, which could lead to unnecessary parental anxiety and healthcare utilization if deployed indiscriminately. The potential of multivariate linear mixture models utilizing iron status and demographic variables to classify both FS risk and recurrence has also been demonstrated (70). However, these models remain exploratory, as their sample sizes were modest (n=162) and replication in independent cohorts is still awaited. In summary, while these predictive models represent progress in FS research, they have notable limitations. Most lack extensive validation across diverse populations, are based on small, single-center cohorts, and haven’t been tested prospectively to show real-world benefits like reduced recurrence or improved quality of life. Integrating various lab and EEG parameters into standard care is also challenging. Thus, these models mainly serve as research tools and require further large-scale validation and practical implementation strategies for routine clinical use. Beyond infection and biomarkers, other modifiable and non-modifiable factors contribute to risk stratification. Breastfeeding, particularly exclusive breastfeeding, has been associated with a protective effect against FS, especially in the first 2.5 years of life (67, 68). Environmental factors such as exposure to cold conditions have been associated with an increased risk of FS hospital admissions (77), though one study found weather conditions less significant than epidemic infections (78). Genetic predisposition plays a crucial role, with pathogenic variants in genes like *SCN1A* and *PCDH19* identified in a subset of children with FS, directly contributing to susceptibility in specific cases (56). Furthermore, maternal factors such as fertility treatment have been associated with a slight increase in the relative risk of FS in offspring (79).

**Figure 1:**
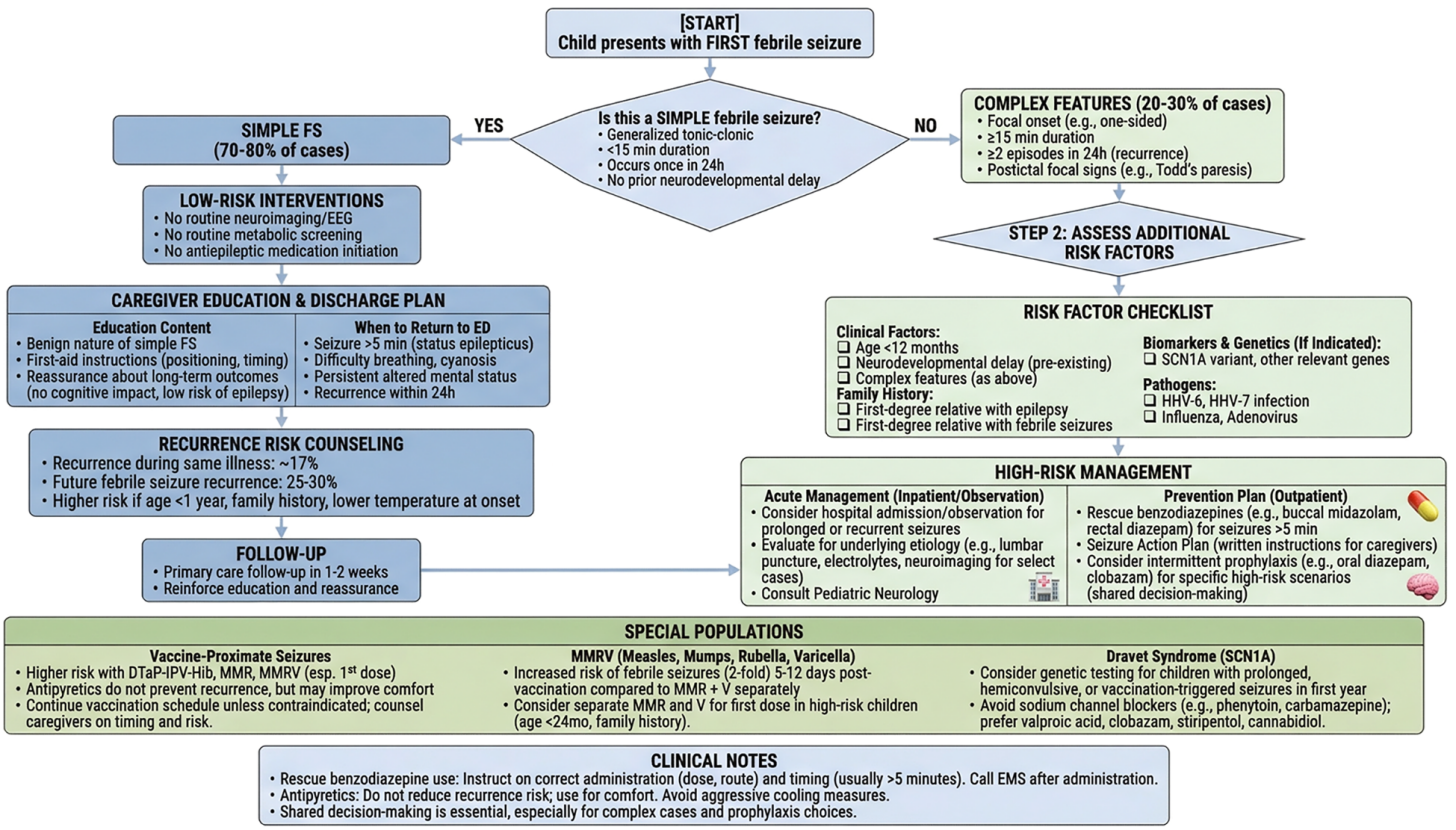
Risk-stratified clinical algorithm for febrile seizure management. This algorithm guides management of children aged 6 months–5 years with first febrile seizure (FS). Simple FS (70–80%) are generalized, <15 min, single in 24h, with return to baseline. Require no routine testing/admission; core intervention is caregiver education on benign prognosis, fever management for comfort, first-aid, return criteria, and recurrence risk (same illness ~17%, future ~25–30%). Complex FS (20–30%) have focal onset, duration ≥15 min, ≥2 episodes/24h, or abnormal exam. Additional risk factors include prolonged >15 min (highest risk), focal features, multiple seizures, age <12 months, developmental delay, family history, low temperature <38.5 °C, hyponatremia <135 mmol/L, and influenza A positivity (OR 3.2). High-risk profile (complex FS plus any additional factor) warrants admission consideration, infection treatment, sodium evaluation, neurology consultation, and rescue benzodiazepine prescription (intranasal midazolam preferred; rectal diazepam alternative). Special populations: vaccine-proximate prolonged seizure (consider SCN1A testing); first-dose MMRV in at-risk children (offer separate MMR+V, reducing FS risk by ~50%); known Dravet syndrome (do not withhold vaccination). Rescue benzodiazepines prescribed in ~12% of FS children. Antipyretics do not prevent distant FS. Shared decision-making essential. FS, febrile seizure; OR, odds ratio; MMRV, measles-mumps-rubella-varicella; MMR+V, measles-mumps-rubella plus varicella given separately.

This evolving landscape of risk stratification, encompassing clinical history, biochemical profiles, inflammatory signatures, and pathogen-specific data, offers the potential for more refined prediction. However, it is important to note that most biomarkers discussed in this section remain investigational, and their clinical utility requires further validation. At present, risk stratification continues to rely primarily on well-established clinical predictors—including age at first seizure, complex features, family history, and recurrence patterns. Nevertheless, these emerging data may help clinicians identify subgroups of children who could be considered for targeted preventive strategies, such as intermittent benzodiazepines during subsequent febrile illnesses, while avoiding unnecessary medication in those at low risk. The ultimate goal is to integrate these evidence-based predictors into practical algorithms that can be applied in diverse clinical settings, from the emergency department to the primary care office, to optimize management and alleviate family anxiety. The corresponding clinical algorithm is presented in **Figure 1**.

## 6 The vaccination conundrum: balancing immunization benefits with febrile seizure risk

The precise prediction of FS risk, as explored in the preceding context, finds one of its most consequential applications in the domain of childhood immunization. Vaccination represents a cornerstone of pediatric preventive medicine, yet its association with fever and, consequently, FS presents a significant clinical and public health dilemma. This conundrum necessitates a nuanced understanding of the attributable risks, which must be weighed against the substantial and well-documented benefits of vaccination in preventing serious infectious diseases and their own, often higher, risks of neurological complications (80). The management of this balance is a critical component of FS prevention strategies, influencing clinical guidelines, parental counseling, and vaccination policies.

The association between certain vaccines and an increased relative risk of FS is established, but it is crucial to emphasize that vaccine-induced febrile seizures are an infrequent event. **Table 4** presents the specific attributable risks across different vaccines, formulations, and dose schedules (80, 82, 83, 88, 89). Notably, the risk profile differs between vaccine formulations. Studies indicate a roughly twofold increased relative risk of febrile convulsions after the first dose of the quadrivalent measles, mumps, rubella, and varicella (MMRV) vaccine compared to the separate administration of MMR and varicella (MMR+V) vaccines at the same visit (88) (88). Importantly, this increased risk is confined to the first dose; the second dose of MMRV does not carry an elevated risk compared to MMR+V or MMR alone, irrespective of the child’s age, sex, history of FS, or the type of vaccine used for the first dose (82). Post-marketing surveillance studies, such as the VigiVax study using SMS-based active monitoring, continue to identify FS following MMRV (89). For other routine vaccines, the 13-valent pneumococcal conjugate vaccine (PCV13) has also been associated with an elevated risk. A large cohort study using Korean national registry data found a transient increased risk of FS following PCV administration, with an incidence rate ratio of 1.27 (84). Similarly, a study within the FDA Sentinel Initiative found an elevated risk after PCV13 (83). This risk may be potentiated by co-administration with inactivated influenza vaccine (IIV), though IIV alone does not appear to independently increase FS risk (83). A randomized trial assessing the strategy of delaying IIV administration by two weeks from DTaP and PCV13 did not reduce the occurrence of post-vaccination fever, suggesting limited utility for this approach in fever prevention (85).

**Table 4:** Vaccines and febrile seizure risk: absolute risks, relative risks, and clinical implications.

Vaccine	Dose	Relative risk (RR/IRR) & source	Absolute risk (attributable risk)	Risk window (days post-vaccination)	Key context/comparison	Clinical recommendation	References
MMR (Measles-Mumps-Rubella)	1st	RR ~2-3 (Cochrane Review). Peak RR 3.36 within 2 weeks.	1 per 1,150 to 1,700 doses (vaccine-attributable).	5–14 days (Peak 7–10 days).	Natural measles infection is a strong trigger for FS and serious complications.	Benefits overwhelmingly outweigh risks. Do not delay vaccination. Administer at 12–15 months when FS risk is lowest.	(9, 80, 81)
MMRV (MMR-Varicella)	1st	RR ~2.0 vs. separate MMR+V (Class effect).	~1 additional FS per 2,500 doses vs. MMR+V.	5–12 days (or 7–10 days).	Varicella (chickenpox) infection itself can cause FS and serious complications.	Balance convenience vs. risk. For children without personal/family history of FS: MMRV is acceptable. FOR children with such history: recommend MMR + V separately for 1st dose to mitigate risk.	(1, 81)
MMRV (MMR-Varicella)	2nd	No significant increase in risk.	Negligible.	Not defined.	—	Safe. No special precautions needed. MMRV is preferred for 2nd dose to complete schedule.	(82)
PCV13 (13-valent Pneumococcal)	Any	IRR 1.27 (95% CI 1.10–1.47). IRR 1.80 (95% CI 1.29–2.52) in another study.	0.33 to 5.16 per 100,000 doses (age-dependent, highest at ~65 weeks).	0–1 days (primary) or 0–7 days.	Pneumococcal disease causes high fever and can lead to seizures, meningitis, and other severe outcomes.	Benefits far outweigh the small, transient risk. Do not delay or withhold. Counsel parents about possible post-vaccination fever.	(83, 84)
PCV13 + IIV (Coadministration)	Any	Possible interaction. IRR for concomitant vaccination 2.80 (95% CI 1.63–4.83) vs. PCV13 alone (IRR 1.54).	Slightly higher than single vaccines.	0–1 days.	Both influenza and pneumococcal infections are common causes of febrile illness and FS in children.	No routine recommendation to separate administrations. IIV alone carries no independent FS risk. The overall benefit of timely vaccination against both diseases is paramount.	(83, 85)
IIV (Inactivated Influenza Vaccine)	Any (Alone)	No significant increase. Adjusted IRR 1.12 (95% CI 0.80–1.56).	Negligible independent risk.	—	Influenza is a very common trigger for FS and causes significant morbidity.	Strongly recommendedPrevents influenza and its complications, including FS.	(83)
COVID-19 mRNA Vaccine	Any	Protective. aOR 0.32 (95% CI 0.18–0.55) for FS vs. unvaccinated.	Reduces FS risk by 68% by preventing infection.	Long-term (via infection prevention).	Omicron infection (BA.5/XBB) was associated with 41.2% FS rate in infants <1 year in one study.	Vaccination significantly reduces the risk of SARS-CoV-2-associated FS, especially in infants.	(86)
RTS, S/AS01E (Malaria Vaccine)	Any	No significant increase reported in large trials.	Comparable to control vaccines.	—	Malaria is a major cause of febrile illness and can provoke complex FS in endemic areas.	Benefits of preventing malaria outweigh potential risks.	(87)

MMR, measles-mumps-rubella; MMRV, measles-mumps-rubella-varicella; PCV13, 13-valent pneumococcal conjugate vaccine; IIV, inactivated influenza vaccine; RR, risk ratio; IRR, incidence rate ratio; aOR, adjusted odds ratio; FS, febrile seizure; CI, confidence interval. Note: Absolute risks are estimates from large observational studies. The protective effect of COVID-19 vaccination is mediated through the prevention of SARS-CoV-2 infection, which itself carries a substantial risk of provoking FS.

The rarity of vaccine-associated febrile seizures must be strongly emphasized when contextualized within the broader epidemiological landscape. While FS occur in 2-4% of all young children from any cause the risk from vaccination is orders of magnitude lower (80). Critically, the risk of FS and far more severe neurological sequelae from the natural diseases vaccines prevent is significantly higher. For instance, the risk of febrile convulsions is estimated at 1 in 43 children with natural measles infection, which is approximately 30 to 40 times greater than the vaccine-attributable risk (88). This stark contrast underscores that vaccination is a net protective measure against febrile seizures provoked by the target pathogens themselves. Similarly, during the Omicron wave of SARS-CoV-2 infection, full vaccination in children was associated with a 68% reduction in the risk of FS compared to being unvaccinated (86). A Danish nationwide cohort study also found a significantly lower risk of FS among vaccinated 5-11-year-olds infected with Omicron compared to their unvaccinated peers (90). For the RTS,S/AS01E malaria vaccine, a meta-analysis of randomized controlled trials showed a comparable overall risk of serious adverse events, including febrile convulsions, between the vaccine and control groups, while demonstrating a protective effect against severe malaria (87). These data underscore that vaccination can be a net protective factor against FS provoked by the target pathogen itself.

A key aspect of this conundrum involves genetic susceptibility. Pathogenic variants in the *SCN1A* gene, which cause Dravet syndrome and are associated with genetic epilepsy with FS plus (GEFS+), can be unmasked by vaccine-proximate FS (91). A prospective study found pathogenic *SCN1A* variants in infants presenting with their first FS proximate to vaccination, with these children later developing Dravet syndrome (91). This indicates that while vaccination does not cause epileptic encephalopathies, it can act as a non-specific trigger for the first seizure in children with an underlying genetic predisposition (81). The discovery of such variants highlights the potential for more personalized management in select high-risk cases, though routine genetic screening for all children with FS is not currently recommended. Therefore, in carefully selected cases—particularly infants with prolonged (≥15 minutes), vaccine-proximate FS, or those with a strong family history of epilepsy or GEFS+—genetic testing for SCN1A and other relevant genes may be considered to inform vaccination planning and seizure management (81, 91). However, such testing should be guided by a specialist and accompanied by appropriate pre- and post-test genetic counseling.

For the broader population of children with a history of SFS or epilepsy, the consensus is clear: vaccination is not contraindicated and should be strongly encouraged (81). Research from China indicates that seizures are often overestimated as a contraindication, leading to unnecessary vaccination deferral (92). A study in Zhejiang province found that 76.1% of children with a history of seizures were recommended to receive vaccines normally, and among those vaccinated as recommended, 88.73% experienced no serious side effects or seizure recurrence (92). This demonstrates the overall safety of proceeding with immunization in this group. Prophylactic antipyretics are not routinely recommended for fever prevention post-vaccination in otherwise healthy children, as there is no evidence they decrease FS risk and they may attenuate the antibody response (81). However, an exception is made for children with Dravet syndrome, where prophylactic antipyretic medication is advised due to their high risk of prolonged seizures with fever (81).

Navigating this conundrum in clinical practice involves clear communication and strategic choices. For the first dose of measles-containing vaccine in measles-naïve infants, offering separate MMR+V injections instead of the combined MMRV product can attenuate the risk of febrile convulsions in predisposed children, such as those with a personal or family history of FS (88). This approach balances individual risk reduction with the population benefits of the combined vaccine, which includes fewer injections and potentially higher coverage rates (88). The overall benefit-risk profile of MMRV remains positive for the general population (88). For other vaccines, adhering to recommended schedules is paramount, as the risks of the diseases far outweigh the transient, small increase in FS risk. Educating both healthcare providers and parents is essential to counteract vaccine hesitancy rooted in unfounded fears of seizures (92). The implementation of vaccine administration laws, as seen in China, can enhance surveillance sensitivity and public confidence by standardizing adverse event reporting, even as reported rates of common reactions like fever increase due to improved detection (93).

Emerging digital technologies for active surveillance, such as the SMS-based system used in the VigiVax study, offer promising tools for more accurately quantifying and communicating these risks in real-world settings (89). Furthermore, exploratory research into genetic factors, such as specific human leukocyte antigen (HLA) types, suggests a possible genetic susceptibility to fever after measles vaccination, which was correlated with a higher measles antibody geometric mean titer (94). While these findings require replication, they point toward future avenues for understanding individual variability in vaccine reactogenicity. The central message remains that the prevention of devastating infectious diseases through vaccination constitutes a primary and highly effective strategy for preventing a vast number of fever-related illnesses and their complications, including FS. The challenge lies in implementing this strategy with precision, ensuring that children at highest genetic risk receive individualized care while maintaining robust, universal immunization coverage to protect the health of all children.

## 7 Emerging frontiers: novel therapeutic targets, mechanisms, and non-pharmacological approaches

**Figure 2:**
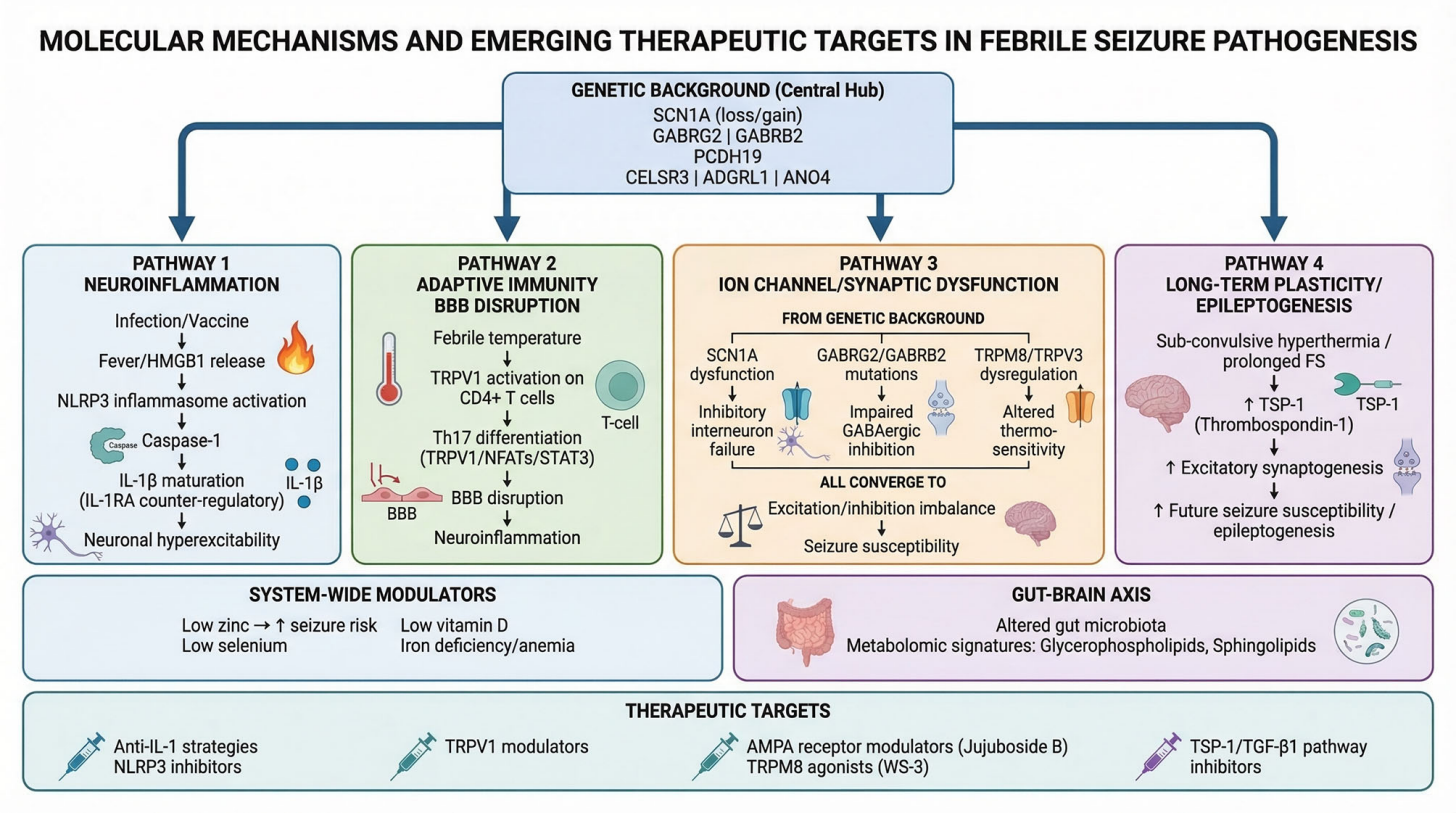
Molecular mechanisms and emerging therapeutic targets in febrile seizure pathogenesis. This diagram integrates genetic, inflammatory, and synaptic mechanisms underlying febrile seizure (FS) susceptibility. Genetic background (SCN1A, GABRG2, GABRB2, PCDH19, CELSR3, ADGRL1, ANO4) modulates all pathways. Pathway 1 (Neuroinflammation): Infection/vaccine triggers HMGB1 release → NLRP3 inflammasome activation → caspase-1 → IL-1β maturation → neuronal hyperexcitability. IL-1RA is endogenous counter-regulator. Pathway 2 (Adaptive Immunity): Febrile temperature activates TRPV1 on CD4+ T cells → Th17 differentiation (TRPV1/NFATs/STAT3) → BBB disruption → neuroinflammation. Pathway 3 (ion channel/synaptic dysfunction): three parallel mechanisms converge: SCN1A dysfunction → inhibitory interneuron failure; GABRG2/GABRB2 mutations → impaired GABAergic inhibition; TRPM8/TRPV3 dysregulation → altered thermosensitivity → excitation/inhibition imbalance → seizure susceptibility. Pathway 4 (long-term plasticity): sub-convulsive hyperthermia ↑ TSP-1 → ↑ excitatory synaptogenesis → ↑ future seizure susceptibility/epileptogenesis (TSP-1/TGF-β1 pathway). System-wide modulators: zinc, selenium, iron, vitamin D deficiencies. Gut-brain axis: altered microbiota, metabolomic signatures. Therapeutic targets organized by pathway: anti-IL-1/NLRP3 inhibitors; TRPV1 modulators; AMPA modulators (Jujuboside B); TRPM8 agonists (WS-3); TSP-1/TGF-β1 inhibitors. Abbreviations: HMGB1, high-mobility group box 1; NLRP3, NOD-like receptor protein 3; IL-1β, interleukin-1β; BBB, blood-brain barrier; Th17, T helper 17 cells; TSP-1, thrombospondin-1; TGF-β1, transforming growth factor beta 1.

The following section discusses emerging mechanisms and novel therapeutic targets based primarily on preclinical studies, animal models, and exploratory human research. These findings are hypothesis-generating and, unless explicitly noted, have not yet reached clinical translation. This section is intended as a roadmap for future research rather than a source of current clinical recommendations. Advancing beyond established pharmacological and management strategies, the frontier of FS research is increasingly focused on deciphering underlying molecular and physiological mechanisms to identify novel therapeutic targets. This shift from a purely symptomatic approach to a mechanism-targeted paradigm is fueled by insights from genetic studies, advanced neuroimaging, systems biology, and sophisticated animal models (51, 95). A critical avenue is the precise elucidation of genetic contributions, extending beyond the well-established SCN1A gene. While SCN1A variants underlie a spectrum from Genetic Epilepsy with Febrile Seizures Plus (GEFS+) to Dravet syndrome, recent work refines genotype-phenotype correlations, showing that variants in crucial functional domains or causing complete loss-of-function are associated with earlier seizure onset and more severe phenotypes (96). Furthermore, novel gene associations are being uncovered, such as CELSR3 with FS and epilepsy with antecedent FS (97), ADGRL1 variants linked to conditions ranging from GEFS+ to developmental and epileptic encephalopathy (98), and ANO4 mutations identified in both sporadic encephalopathic and familial fever-sensitive epilepsies (99). Importantly, functional divergence in known genes, such as gain-of-function versus loss-of-function variants in GABRB2, is now linked to distinct clinical severities, with loss-of-function more associated with fever-triggered seizures and milder outcomes (100). This genetic dissection not only aids in prognosis and diagnosis but also directly points to specific neuronal pathways—sodium channels, GABAergic inhibition, and calcium-activated processes—as targets for precision therapeutics (101, 102). The major pathogenic pathways are schematized in **Figure 2**.

Central to the pathophysiology of FS is neuroinflammation, which represents a rich domain for intervention. Evidence consistently points to an amplified inflammatory response during FS episodes compared to febrile episodes without seizures (11). Key mediators include High-Mobility Group Box 1 (HMGB1), a danger-associated molecular pattern whose levels are elevated in FS, correlate with seizure complexity and duration, and are associated with progression to epilepsy (10). HMGB1 is implicated in activating the NLRP3 inflammasome, leading to Caspase-1 activation and the release of pro-inflammatory cytokines like IL-1β (13). The IL-1 axis appears particularly relevant, with elevated IL-1RA levels observed during FS (11), and genetic polymorphisms in the IL-1β gene showing association with FS susceptibility, albeit distinct from other fever-induced encephalopathies (103). Conversely, reduced levels of the anti-inflammatory cytokine IL-10 and specific IL-10 gene polymorphisms are linked to increased FS risk (12). This inflammatory cascade offers multiple nodes for intervention. Research in animal models demonstrates that targeting these pathways can be effective. For instance, the traditional herb Tetrastigma hemsleyanum was shown to suppress neuroinflammation and exert anticonvulsant effects by inhibiting the PKC-δ/caspase-1 signaling pathway (104). Another critical link is between fever, adaptive immunity, and neuroinflammation; recent work reveals that febrile temperatures activate TRPV1 on CD4+ T cells, promoting Th17 cell differentiation via the TRPV1/NFATs/STAT3 pathway, which subsequently disrupts the blood-brain barrier (BBB) and exacerbates neuroinflammation in complex FS models (105). These findings identifies a mechanistic pathway that may be targetable in future research for preventing seizure recurrence or mitigating long-term consequences.

Concurrently, research is illuminating the roles of specific ion channels and synaptic mechanisms in hyperthermia-induced neuronal hyperexcitability. Beyond sodium and GABA receptors, thermosensitive Transient Receptor Potential (TRP) channels are emerging as key molecular thermostats. The TRPM8 agonist WS-3 was shown to suppress hyperthermia-induced FS and fast ripple activity in infant mice without altering temperature thresholds, whereas TRPM8 deficiency lowered these thresholds and worsened seizures (106). Conversely, TRPV3 appears to play a role in maintaining cortical network stability during fever-range temperatures; its activity helps a subset of neurons stay active, and its absence delays seizure onset, suggesting a complex, homeostatic function rather than a purely pro-convulsant one (107). Synaptic targets are also under investigation. Jujuboside B, a compound from traditional medicine, was found to alleviate FS by inhibiting AMPA receptor-mediated currents and neuronal excitability (108). Furthermore, research highlights that even hyperthermia below the seizure threshold (sub-FS stimuli) can have lasting consequences by upregulating Thrombospondin-1 (TSP-1), promoting excitatory synaptogenesis, and thereby increasing future seizure susceptibility; inhibiting the TSP-1/TGF-β1 pathway mitigated these effects (109). These studies underscore that interventions need not solely target the acute convulsion but could also aim to modify the neural circuitry’s long-term plasticity induced by febrile stimuli.

Systems biology approaches, including metabolomics and microbiome analysis, are uncovering novel biomarkers and systemic alterations associated with FS. Metabolomic profiling of plasma from children with FS reveals distinct disturbances in pathways such as glycerophospholipid metabolism, sphingolipid signaling, and necroptosis (110). Specific metabolites, including arachidonic acid, various lysophosphatidylcholines, and sphingomyelins, have been identified as potential diagnostic biomarkers (110, 111). Parallel analyses of gut microbiota show reduced α-diversity in FS patients and altered abundances of specific bacterial genera, suggesting a possible gut-brain axis involvement in FS pathogenesis (111). These “omics” signatures not only offer tools for risk stratification but also hint at broader systemic metabolic or inflammatory states that predispose to seizures, generating hypotheses for future nutritional or microbiome-based studies. Furthermore, proteomic analysis of the BBB in adult mice that experienced complex FS in infancy revealed lasting alterations, particularly in the extracellular matrix (ECM)-receptor interaction pathway, which was linked to increased epilepsy susceptibility in adulthood (112). This points to the BBB and its associated pathways as potential targets for preventing epileptogenesis following severe FS.

Non-pharmacological and technological innovations are also emerging. The recognition of a strong circadian pattern in FS occurrence, with a peak in the late afternoon and early evening, introduces the concept of chronotherapy (113). This knowledge could guide the timing of prophylactic measures for high-risk children. In the realm of acute management and monitoring, wearable sensor technology is advancing rapidly. For instance, hollow-structured composite fibers enabling simultaneous high-sensitivity pressure and humidity sensing have been integrated into an early alarm system to monitor muscle activity and breathing rate for real-time detection of febrile convulsions (114). While earlier attempts at non-invasive acute interventions, such as home-use carbogen (CO2) inhalation, did not prove more effective than oxygen placebo in stopping seizures (115), they underscore the ongoing search for accessible, non-sedating abortive methods. Finally, the optimization of traditional herbal formulations through modern analytical techniques represents a convergence of empirical knowledge and contemporary science. Comparative studies show that traditionally processed Zixue Powder retains higher levels of active constituents, exhibits faster *in vitro* release, and demonstrates superior efficacy in prolonging seizure latency and reducing neuronal damage in animal models compared to its pharmacopoeial counterpart (116). Similarly, the mechanistic elucidation of herbs like Saiga horn, which appears to exert anticonvulsant effects by upregulating the brain’s serotonergic pathway and suppressing neuroinflammation (117), offers preliminary mechanistic insights that require validation.

The exploration of early-life origins and long-term neural sequelae further refines potential intervention windows. Studies in animal models demonstrate that experimental FS lead to sustained alterations in the glutamatergic system of hippocampal-prefrontal circuits, with region-specific neuronal loss and downregulation of glutamate receptors and transporters, correlating with behavioral deficits like anxiety in adulthood (118). Even in the absence of behavioral seizures, hyperthermia can alter synaptogenesis and increase future seizure susceptibility via pathways involving TSP-1 (109). Remarkably, research suggests that the long-term cognitive impairments following prolonged FS in rat pups may be linked not to the behavioral seizure itself but to a subsequent phase of occult epileptiform activity and associated cerebral hyperoxia (95). Furthermore, epidemiological research hints at prenatal origins, with alterations in placental gene expression related to glucocorticoid and serotonergic systems being associated with FS incidence and earlier age of onset in offspring (119). These findings collectively argue for a life-course perspective on FS prevention, where strategies could span from optimizing the prenatal environment to implementing post-seizure neuroprotective interventions aimed at preventing maladaptive plasticity and epileptogenesis.

In summary, while the mechanisms and targets discussed above offer exciting avenues for future investigation, they remain largely experimental. Their clinical application awaits rigorous validation in prospective human studies and clinical trials.

## 8 Empowering caregivers: education, psychological support, and home management as preventative tools

Transitioning from a life-course perspective that identifies potential intervention windows, the immediate and practical burden of FS management falls predominantly on caregivers. Effective prevention and harm reduction strategies must therefore extend beyond clinical settings to empower families. The role of caregivers as frontline responders is not only pivotal in acute management but also constitutes a critical preventative layer through informed home care, appropriate emergency response, and psychological resilience, which can mitigate anxiety-driven behaviors and reduce unnecessary healthcare utilization.

A foundational barrier to effective caregiver management is a widespread lack of accurate knowledge about FS. Studies consistently reveal significant knowledge gaps among parents, irrespective of whether their child has experienced an FS. Research indicates that over half of parents had not informed themselves about FS prior to an event, and only 32% of parents realized their child was having an FS when the first seizure occurred (120). This lack of knowledge is a global concern, with mothers of children with a history of FS demonstrating poor FS knowledge, which is significantly associated with higher levels of state and trait anxiety and uncertainty (121). This phenomenon of “fever phobia” is pervasive, with surveys showing that FS is the most feared complication of fever, reported by 73.1% of mothers, leading to high parental anxiety scores and often inappropriate fever management practices such as administering antipyretics at short intervals (122). The psychological impact on caregivers during and after an FS event is profound and constitutes a significant aspect of the FS burden. The majority of parents describe intense fear during their child’s seizure, with a median intensity rated as 10 out of 10 (120). This acute stress can evolve into sustained anxiety, depression, and stress, particularly around the time of hospital admission. A study in Malaysia found high prevalence rates of anxiety (58.2%), stress (29%), and depression (23.6%) among parents at the time of their child’s admission for FS (35). Factors such as younger child age and longer hospital stay were associated with increased parental anxiety (35). The experience is traumatizing, leading 77% of affected parents to report they will observe their child more carefully after the first event, and 63% stating they will give antipyretics earlier, at a median temperature of 38.2 °C (120). This anxiety-driven hypervigilance and altered fever management underscore the need for targeted psychological support and education to prevent maladaptive coping strategies.

Structured educational and psychological interventions have demonstrated significant efficacy in improving outcomes for both children and their families (34, 36–38). Nurse-led empowerment programs have shown promise in transforming caregiver competence. A quasi-experimental study in Egypt implementing a Febrile Convulsion Empowerment Program for mothers resulted in a dramatic improvement, shifting from 75% reporting poor knowledge pre-intervention to 85.2% reporting good knowledge post-intervention. Similarly, unsatisfactory home management practices improved from 66.5% to 81.4% after the intervention, with a strong positive correlation found between knowledge and practice (36). Beyond knowledge transfer, interventions addressing psychological needs yield tangible clinical benefits. A study evaluating nursing interventions based on the Kano model, which tailors care to patient and family needs, found that children in the intervention group had less frequent FS recurrence, shorter times to cessation of convulsions and fever reduction, and improved psychological behavior scores compared to a general care group (34). Furthermore, the parents in the Kano group exhibited lower perceived stress and overall psychological symptom scores, alongside higher satisfaction (34). Similarly, combining targeted emergency nursing with psychological nursing for children with FS led to faster resolution of fever and convulsions, shorter hospital stays, and significantly lower parental anxiety and depression scores, alongside higher treatment compliance and satisfaction (37). The importance of structured follow-up is highlighted by a prospective randomized study, which, while focused on first unprovoked seizures, provides a comparative framework. It showed that a timely, semi-structured follow-up in a first seizure clinic led to a significant and sustained reduction in parental and child anxiety levels compared to delayed follow-up. Notably, for the FS group followed without a specific intervention, parental state anxiety also decreased significantly within three months, suggesting a natural resolution over time but also indicating room for accelerated recovery through support (38).

To tailor education effectively, reliable tools are needed to assess caregiver knowledge. The translation and validation of the Febrile Convulsion Knowledge Scale for Parents/Caregivers into Chinese provides a psychometrically sound instrument with good reliability and validity (123). Such tools enable healthcare professionals to accurately identify knowledge gaps and design targeted educational plans, moving beyond generic advice to personalized guidance. The sources from which caregivers seek information also critically influence their knowledge and anxiety. An analysis of YouTube videos on FS in Korean revealed that both the quality and reliability of content were generally low, with only about 30% classified as high quality. Videos featuring physicians as the main speaker and longer videos tended to be of higher quality (124). This underscores a responsibility for healthcare professionals to be aware of the prevalence of misinformation on social media, to guide parents toward reliable resources, and to actively create high-quality educational content themselves.

The need for education extends beyond parents to other caregivers involved in child supervision. A study of Korean childcare providers found unfavorable levels of knowledge, attitudes, concerns, and recommended practices regarding FS. Their management practices were significantly related to their knowledge level, prior education on FS, attitudes, and actual experience with FS events (125). This finding emphasizes that FS preparedness must be integrated into childcare provider training programs to ensure a consistent and appropriate response across care settings. Furthermore, effective communication must be culturally competent, as purely biomedical educational messages may fail if they do not engage with local beliefs and socio-cultural frameworks (126). Culturally sensitive health education is essential, particularly in diverse or resource-limited settings.

The consensus on what constitutes core, reassuring information to deliver to families after an FS has been formalized through a Delphi process involving European child neurologists and pediatricians. The resulting key messages aim to reassure caregivers based on epidemiology, underlying mechanisms, and emergency management guidance for potential recurrence (33). This structured approach to post-event counseling is a fundamental preventative tool, equipping families with the understanding needed to manage future febrile illnesses with less fear and more confidence. Ultimately, empowering caregivers with knowledge and support has direct implications for clinical pathways. Evidence suggests that for children with simple FS who are stable and have returned to neurological baseline, an extended observation period in the emergency department may not be warranted, as seizure recurrence is low (1.9%) and typically occurs within the first 3 hours (29). Safe early discharge is contingent upon providing appropriate caregiver education and ensuring follow-up, shifting the focus of “observation” from the hospital to the informed home environment. This paradigm reinforces the caregiver’s role as the central agent in a comprehensive, prevention-oriented management strategy for FS.

## 9 Beyond the febrile illness: long-term outcomes and progression to epilepsy

**Figure 3:**
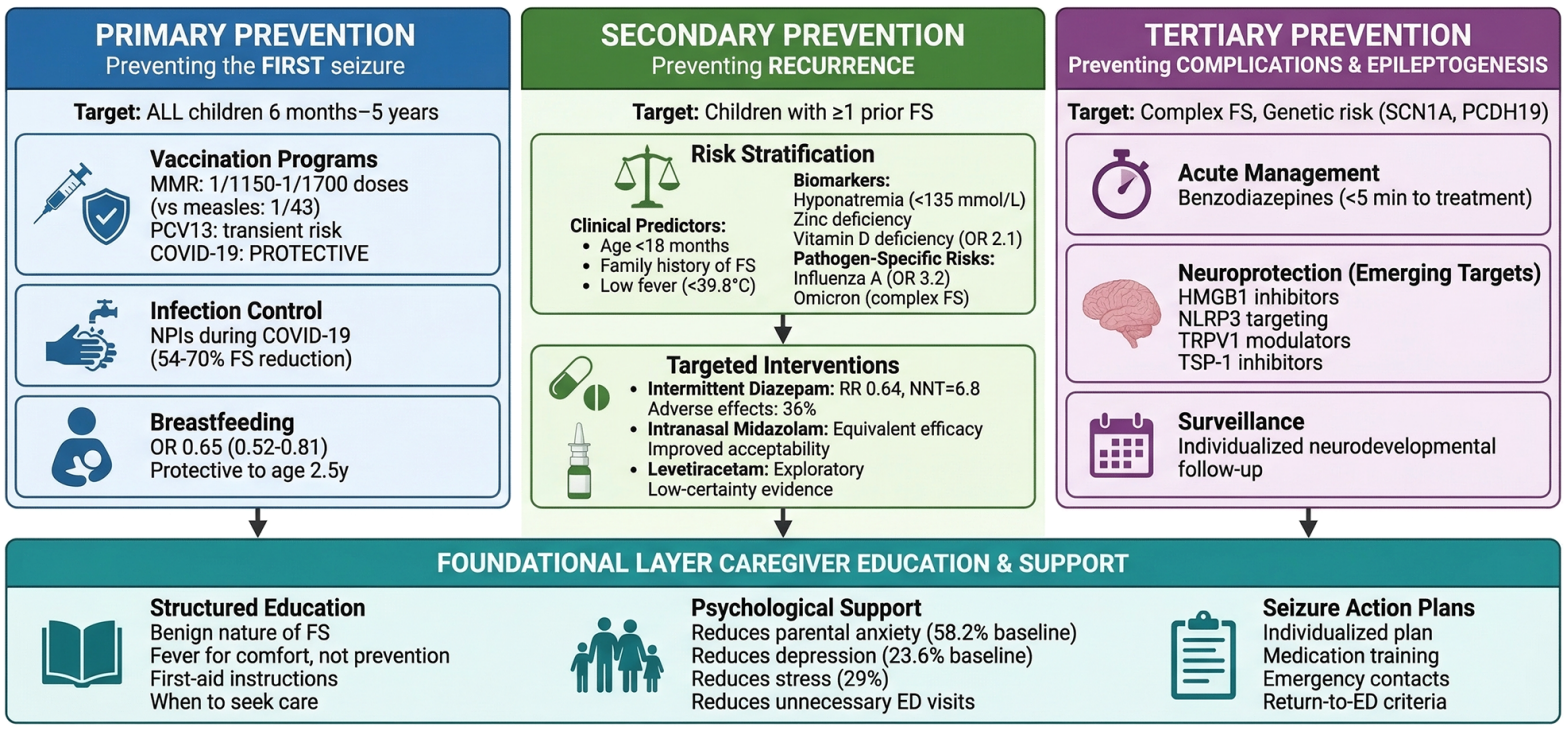
Three-level framework for febrile seizure prevention. This framework integrates evidence into a stratified approach to febrile seizure (FS) prevention. Primary Prevention targets all children aged 6 months–5 years to prevent first FS. Interventions include vaccination (MMR: 1/1150–1/1700 dose risk vs measles 1/43; PCV13 transient risk; COVID-19 protective), infection control (NPIs reduced FS by 54–70%), and breastfeeding (OR 0.65, protective to age 2.5 years). Secondary Prevention targets children with ≥1 prior FS to prevent recurrence. Risk stratification integrates biomarkers (hyponatremia, zinc/vitamin D deficiency [OR 2.1]) and pathogen-specific risks (influenza A [OR 3.2], Omicron). Targeted interventions for high-risk children include intermittent diazepam (RR 0.64, NNT = 6.8, adverse effects 36%), intranasal midazolam (equivalent efficacy), and levetiracetam (exploratory). Tertiary Prevention targets children with complex FS or genetic predisposition (SCN1A, PCDH19) to prevent complications. Strategies include rapid benzodiazepines, emerging neuroprotective targets (HMGB1, NLRP3, TRPV1, TSP-1), and neurodevelopmental surveillance. Foundational Layer: Caregiver Education addresses benign nature, fever management, first-aid, and return criteria, reducing anxiety (58.2% baseline) and healthcare utilization. Abbreviations: FS, febrile seizure; MMR, measles-mumps-rubella; PCV13, 13-valent pneumococcal vaccine; NNT, number needed to treat; RR, risk ratio; OR, odds ratio; HMGB1, high-mobility group box 1; NLRP3, NOD-like receptor protein 3; TRPV1, transient receptor potential vanilloid 1; TSP-1, thrombospondin-1.

The reassuring narrative of FS as a benign, self-limited phenomenon, which forms the cornerstone of caregiver counseling, is statistically valid for the overwhelming majority of children. However, a crucial aspect of long-term management and prevention research involves understanding and mitigating the small but significant risk of adverse neurological sequelae. The primary concern for families and clinicians alike is whether a FS heralds the onset of chronic epilepsy. Extensive epidemiological data confirm that while most children outgrow FS without sequelae, a subset is at increased risk for developing unprovoked seizures later in life. This risk is not uniform and is strongly stratified by specific clinical features, guiding prognostication and long-term follow-up strategies (51). The three-level prevention framework for FS is summarized in **Figure 3**.

Children with SFS have an epilepsy risk of approximately 1-2%, only marginally higher than the general population. In contrast, children with CFS face a substantially elevated risk of 4-15%, depending on the specific complex features present (51).FS. Prolonged FS, in particular, have been a focus of research due to their association with mesial temporal lobe epilepsy and hippocampal sclerosis in some individuals, suggesting a potential causal link in susceptible brains (51). The baseline risk of multiple seizures within the same febrile illness, a hallmark of CFS, is estimated at 17%, with significant heterogeneity across studies (44). This risk underscores the clinical scenario that often triggers the most anxiety and the consideration of acute abortive therapies.

Beyond the index seizure characteristics, other factors modulate the long-term epilepsy risk. A family history of epilepsy (not just FS), pre-existing neurological abnormalities or developmental delay, and the occurrence of FS at a very young age (especially <12 months) are all recognized contributors to an elevated risk profile. The presence of multiple risk factors compounds the likelihood. Recent research continues to explore the intricate pathogenic interplay of fever, genetic susceptibility, and neuroinflammation that may lower the seizure threshold not only acutely but also contribute to long-term epileptogenesis (51). The immature, rapidly developing brain’s response to hyperthermia and inflammatory cytokines may, in predisposed individuals, create lasting molecular and structural changes that facilitate future unprovoked seizures.

This delineation of risk has profound implications for prevention strategies, particularly pharmacological ones. The central question is whether aggressive intervention to prevent recurrent FS can alter the long-term trajectory towards epilepsy. The current evidence suggests that for the vast majority of children, particularly those with SFS, pharmacological prophylaxis does not reduce the risk of later epilepsy and introduces its own set of risks (32). Critically, no pharmacological intervention—including intermittent benzodiazepines or continuous antiepileptic drugs—has been shown to reduce the long-term risk of epilepsy. The updated Cochrane review reaffirms that while intermittent diazepam and continuous phenobarbital are effective in reducing the recurrence of FS, their use is associated with adverse effects in a significant proportion of children—up to 30% for phenobarbital and 36% for benzodiazepines (32). More critically, the review concludes that given the benign nature of recurrent FS for most, the risk-benefit calculus does not favor routine prophylaxis, and the focus should remain on caregiver education and support (32). As detailed in Section 4, rescue benzodiazepines reduce short-term recurrence but do not prevent epilepsy.

Ultimately, the long-term management of FS is an exercise in risk communication and strategic preparedness. For the child with SFS and no other risk factors, the most powerful preventative tool against parental anxiety and unnecessary medical intervention is clear, evidence-based counseling on the excellent prognosis. For the child with complex features or other risk factors, management involves vigilant monitoring for recurrent prolonged seizures, a tailored plan for rescue medication use if indicated, and appropriate neurological follow-up to monitor development. The research frontier lies in better identifying the specific genetic, imaging, or biomarker signatures that distinguish the child whose FS is an isolated benign event from one in whom it is the first manifestation of a chronic epileptic disorder. Until such precision is achieved, a nuanced approach that acknowledges the spectrum of long-term risk—without medicalizing the benign majority—remains the standard of care.

EEG is not indicated for children with SFS who are neurologically healthy. It may be considered in CFS, recurrent febrile seizures, cases with abnormal neurological status, a family history of epilepsy, or suspected encephalitis (127). Brain MRI is indicated when focal neurological signs are present, seizures are prolonged (>15 minutes), or a structural abnormality is suspected; routine neuroimaging is not recommended for SFS(17, 127). In the context of prolonged febrile seizures or febrile status epilepticus, acute MRI findings carry important prognostic value. Approximately 27% of children show unilateral hippocampal hyperintensity on diffusion-weighted imaging (DWI) within days of febrile status epilepticus, suggesting cytotoxic edema (128). This finding is strongly associated with subsequent epilepsy: 100% of children with acute DWI hyperintensity developed focal epilepsy over 9–13 years of follow-up, compared to only 10% of those without such findings (128). The FEBSTAT study further found that acute hippocampal T2 hyperintensity predicted hippocampal sclerosis and a 22% 10-year cumulative incidence of mesial temporal lobe epilepsy (129). However, these findings should be interpreted with caution. First, the sample sizes in these prospective studies are relatively small (128), which limits the generalizability of the observed associations. Second, the relationship between hippocampal sclerosis and mesial temporal lobe epilepsy is not always linear: in the FEBSTAT study, only 2 of 6 subjects who developed MTLE had definite hippocampal sclerosis, while 2 had equivocal and 2 had no sclerosis (129), suggesting that factors beyond structural MRI findings contribute to epileptogenesis. For follow-up, children with SFS generally require no specialized neurological follow-up beyond reassurance and standard pediatric care. In contrast, those with CFS, abnormal neuroimaging findings, or additional risk factors—such as developmental delay or a strong family history of epilepsy—should be referred to pediatric neurology for long-term surveillance (127, 130).

## 10 Conclusions and Future Directions: Integrating Evidence into Risk-Stratified Prevention Approaches.

The accumulated evidence and evolving clinical practices delineated in this review converge on a central paradigm shift in FS management: moving from a uniform, often medication-centric approach for all children towards a nuanced, risk-stratified, and personalized prevention strategy. This evolution is informed by a clearer understanding of the distinct recurrence risks within the same febrile illness versus across distant episodes, the variable efficacy and safety profiles of available interventions, and the critical importance of caregiver context. The foundation of modern prevention no longer rests on the routine pharmacological suppression of all FS but on the strategic integration of accurate prognosis, targeted pharmacological rescue for high-risk scenarios, and comprehensive caregiver empowerment.

It is important to emphasize that, at present, no validated biomarker-based or immunologically guided strategy is available for routine clinical prevention of FS. Clinical decision-making still relies primarily on established clinical risk factors—such as age at first seizure, complex features, family history, and recurrence patterns—and intermittent benzodiazepines remain the only pharmacological approach supported by moderate-quality evidence for selected high-risk patients. The concept of “personalized prevention” as discussed in this review therefore refers to risk-stratified application of existing tools rather than biomarker-driven precision medicine, which remains a future research goal.

Current prophylactic strategies are defined by their specific indications and limitations. The evidence firmly establishes that antipyretics, whether acetaminophen or ibuprofen, have no role in preventing FS recurrence during distant fever episodes (32, 39). Any potential benefit appears confined to the same fever episode, and even here, the evidence is limited and requires further validation (39). In contrast, intermittent benzodiazepine therapy, particularly with diazepam, demonstrates efficacy in reducing recurrence, with moderate-certainty evidence supporting its use over periods of 6 to 24 months (32). This benefit extends specifically to preventing seizure recurrence within the same febrile illness, as shown by studies where rectal diazepam administration significantly reduced short-term recurrence compared to antipyretics alone or no prophylaxis (40, 45). However, this pharmacological benefit is counterbalanced by a significant burden of adverse effects, observed in over a third of children in some studies, and the potential for negative neurocognitive impacts with continuous phenobarbital (32). Consequently, the most recent Cochrane Review concludes that given the generally benign nature of recurrent FS, the decision to use these drugs must weigh their moderate preventative benefit against their frequent and sometimes serious side effects, favoring instead robust caregiver education and support (32).

This cost-benefit analysis naturally leads to the imperative of risk stratification. The baseline risk of multiple seizures within the same febrile illness is a cornerstone for clinical counseling and study design, with a meta-analysis estimating this risk at approximately 17% (44). Not all children bear this risk equally. Key factors identifiable at the initial presentation strongly predict which children are more likely to receive and potentially benefit from rescue medication prescriptions. A CFS and an initial inpatient hospitalization are the factors most strongly associated with a subsequent prescription for a home rescue benzodiazepine (46). Furthermore, an eventual diagnosis of epilepsy is a major independent factor linked to such prescriptions, highlighting that rescue medication strategies often extend beyond the FS period into the management of established epilepsy risk (46). Therefore, personalized prevention hinges on identifying these high-risk features—complex or prolonged initial seizure, family history of epilepsy, neurodevelopmental concerns, or very young age at first episode—to tailor interventions rather than applying them universally.

Parallel to refined risk assessment, the mode of intervention is undergoing a significant transformation. The historical reliance on rectal diazepam is being rapidly supplanted by intranasal formulations of midazolam and diazepam, driven by considerations of social acceptability, ease of administration, and speed (42, 43). This transition from rectal to intranasal rescue benzodiazepines has been progressive and now involves approximately half of patients with repeated prescriptions in some cohorts, though it is associated with increased drug costs (42). This shift in administration route is a key component of personalized care, as it allows for the selection of a formulation that aligns with a specific family’s capabilities, preferences, and the local healthcare infrastructure’s support systems. The choice of agent and route must be individualized, considering factors such as the child’s age (with different FDA approvals for intranasal sprays), the expected need for repeated dosing, and the family’s comfort with administration (43).

Future directions in FS prevention are poised to extend beyond optimizing existing benzodiazepine delivery. Emerging research into pathogenesis points to novel therapeutic targets. The interplay between fever, neuroinflammation, genetic susceptibility, and even the gut-brain axis offers potential avenues for intervention that move upstream from seizure termination to modulation of the hyperexcitable state that predisposes to FS (51). Agents with anti-inflammatory properties or those targeting specific ion channels implicated in genetic epilepsies associated with FS may inform future hypothesis-driven research. Furthermore, the demonstrated efficacy and favorable safety profile of intermittent oral levetiracetam in one trial, though based on very low-certainty evidence, suggests that other anti-seizure medications with different mechanisms of action warrant further investigation in high-risk populations (32).

Ultimately, integrating this evidence into clinical practice requires a holistic, patient-centered framework. The economic impact of management choices, from emergency department visits to drug costs, is a practical consideration for healthcare systems and families (40, 42). The goal is to construct a personalized prevention paradigm that balances the objective recurrence risk (44), the efficacy and tolerability of available rescue therapies (32, 40), the evolving landscape of drug formulations (42, 43), and the subjective burden on the family, including anxiety and quality of life. For the vast majority of children with simple FS, the most effective and safest “prevention” remains confident reassurance, clear first-aid instructions, and guidance on when to seek medical attention. For the minority at higher risk of recurrence, prolonged seizures, or subsequent epilepsy, this paradigm offers a structured approach involving risk factor identification, a shared decision-making process regarding rescue medication prescription, training in the use of an appropriate formulation, and integrated neurological follow-up. The future of FS prevention lies not in a single drug or policy, but in this sophisticated integration of epidemiology, therapeutics, and personalized care planning.
